# Antigen-specific T helper cells and cytokine profiles predict intensity and longevity of cellular and humoral responses to SARS-CoV-2 booster vaccination

**DOI:** 10.3389/fimmu.2024.1423766

**Published:** 2024-08-29

**Authors:** Lukas Page, Kevin Dennehy, Katharina Mueller, Philipp Girl, Eva Loell, Hellen Buijze, Johanna-Maria Classen, Helmut Messmann, Christoph Roemmele, Reinhard Hoffmann, Sebastian Wurster, Andre Fuchs

**Affiliations:** ^1^ Institute for Laboratory Medicine and Microbiology, University Hospital of Augsburg, Augsburg, Germany; ^2^ Bundeswehr Institute of Microbiology, Munich, Germany; ^3^ Central Institute of the Bundeswehr Medical Service, Munich, Germany; ^4^ Institute for Infectious Diseases and Zoonoses, Department of Veterinary Sciences, Faculty of Veterinary Medicine, Ludwig Maximilians University Munich, Munich, Germany; ^5^ Internal Medicine III – Gastroenterology and Infectious Diseases, University Hospital of Augsburg, Augsburg, Germany; ^6^ Department of Infectious Diseases, Infection Control, and Employee Health, The University of Texas MD Anderson Cancer Center, Houston, TX, United States

**Keywords:** COVID-19, immunity, memory cells, immune monitoring, antibodies, cytokines, transcriptomics

## Abstract

**Introduction:**

Pre-existent pools of coronavirus-specific or cross-reactive T cells were shown to shape the development of cellular and humoral immune responses after primary mRNA vaccination against SARS-CoV-2. However, the cellular determinants of responses to booster vaccination remain incompletely understood. Therefore, we phenotypically and functionally characterized spike antigen-specific T helper (Th) cells in healthy, immunocompetent individuals and correlated the results with cellular and humoral immune responses to BNT162b2 booster vaccination over a six-month period.

**Methods:**

Blood of 30 healthy healthcare workers was collected before, 1, 3, and 6 months after their 3rd BNT162b2 vaccination. Whole blood was stimulated with spike peptides and analyzed using flow cytometry, a 13-plex cytokine assay, and nCounter-based transcriptomics.

**Results:**

Spike-specific IgG levels at 1 month after booster vaccination correlated with pre-existing CD154+CD69+IFN-γ+CD4+ effector memory cells as well as spike-induced IL-2 and IL-17A secretion. Early post-booster (1-month) spike IgG levels (r=0.49), spike-induced IL‑2 (r=0.58), and spike-induced IFN‑γ release (r=0.43) correlated moderately with their respective long-term (6-month) responses. Sustained robust IgG responses were significantly associated with S-specific (CD69+±CD154+±IFN-γ+) Th-cell frequencies before booster vaccination (p=0.038), especially double/triple-positive type-1 Th cells. Furthermore, spike IgG levels, spike-induced IL‑2 release, and spike-induced IFN‑γ release after 6 months were significantly associated with increased IL‑2 & IL‑4, IP‑10 & MCP1, and IFN‑γ & IP‑10 levels at 1 month post-booster, respectively. On the transcriptional level, induction of pathways associated with both T-cell proliferation and antigen presentation was indicative of sustained spike-induced cytokine release and spike-specific IgG production 6 months post-booster. Using support vector machine models, pre-booster spike-specific T-cell frequencies and early post-booster cytokine responses predicted sustained (6-month) responses with F1 scores of 0.80-1.00.

**Discussion:**

In summary, spike-specific Th cells and T-cellular cytokine signatures present before BNT162b2 booster vaccination shape sustained adaptive cellular and humoral responses post-booster. Functional T-cell assays might facilitate early identification of potential non-responders.

## 1 Introduction

Since the beginning of the Coronavirus disease 2019 (COVID-19) pandemic, we witnessed a uniquely fast and successful development of vaccines against severe acute respiratory syndrome coronavirus-2 (SARS-CoV-2). Extensive evidence suggests that booster vaccination, i.e., additional vaccine doses following primary immunization, provides enhanced protection against death or severe COVID-19 (1). However, as the virus is continuously evolving, breakthrough infections (BTIs) following vaccination have become more frequent, given the emergence of immune escape variants of SARS-CoV-2 (2–4).

Many conditions associated with suppression of the adaptive immune response can considerably hamper the effect of COVID-19 vaccination (5, 6). However, the relative contributions of the different components of the adaptive immune system to vaccine-induced immune protection, including the determinants of long-term immunity after booster vaccination, remain incompletely understood. In particular, the role of pre-existing antigen-specific T-helper (Th) cells remains to be fully defined in the context of booster vaccination.

Even though pre-existing, seasonal coronavirus-specific T cells can confer some protection against SARS-CoV-2 infection (7), these cells do not seem to be a major contributor to the development of immunity against SARS-CoV-2 after infection or vaccination (8). Instead, pre-existing memory T cells reacting to the SARS-CoV-2 spike protein (S) might even impair the development of a varied T-cell and neutralizing antibody response after the primary BNT162b2 vaccination series (8). This effect has been shown to be age-dependent, as elderly vaccinees have developed a diverse coronavirus-specific T-cell repertoire over their lifetime. These cells cross-react with SARS-CoV-2 epitopes, albeit with low avidity (9, 10). Thus, this response does not mature or diversify into higher-avidity antigen receptor variants and cannot initiate high-quality responses (8). On the other hand, pre-existing S-reactive T cell populations in younger vaccinees exhibit naïve phenotypes (CCR7^+^CD45RA^+^), have high plasticity, and can initiate diverse and high-quality responses, i.e., consistent production of neutralizing antibodies and cytokines after primary immunization (8). Cytotoxic (CD8^+^) T-cell responses were shown to vary considerably between individuals and most vaccinees develop only low frequencies of SARS-CoV-2 specific CD8^+^ T cells following the first three vaccinations (11).

Significant variation in the robustness and longevity of antibody and T-cell responses to booster vaccination against SARS-CoV-2 has been extensively documented (12–17). However, the determinants of sustained adaptive immune responses to booster vaccination against SARS-CoV-2, including the role of T cells present pre-booster, remain scarcely studied. Therefore, we phenotypically and functionally characterized S-specific Th cells in healthy, immunocompetent individuals and correlated the results with cellular and humoral immune responses to BNT162b2 booster vaccination over a six-month period. Thereby, we identified S-specific Th cell populations integral to short-term T-cell expansion, along with cytokine profiles in response to S peptides that correlate with longevity of key antiviral effector functions. These insights might help to identify potential low-responders to SARS-CoV-2 booster vaccination.

## 2 Methods

### 2.1 Subjects and blood collection

As part of an ongoing registry study (Corona Registry Study number 20–426, approved by the ethics committee of the Ludwig Maximilians University Munich), thirty healthy volunteers were recruited among employees of the University Hospital of Augsburg. All subjects had received primary vaccination with two doses of BNT162b2 (Pfizer Inc., New York, NY, USA and BioNTech SE, Mainz, Germany) and were enrolled before their third dose of BNT162b2 (first booster). Exclusion criteria were active infections, receipt of immunomodulatory therapy, pregnancy, positive nucleocapsid-specific (N) IgG titers, and underlying conditions associated with significant impairment of anti-COVID-19 immunity (e.g., diabetes mellitus, cancer, prior stem cell transplantation, or autoimmune diseases). Lithium heparin-anticoagulated whole blood and serum samples were collected immediately before the third vaccination (T0), as well as 1 (T1), 3 (T3), and 6 months (T6) after booster vaccination (**Supplementary Figure S1**). Demographic data of study participants are summarized in **Supplementary Table S1** for each individual timepoint. No additional booster doses were given during the study period.

### 2.2 Antibody measurement

Serum was tested for N- and S-specific IgG using a COBAS e801 immunoassay analyzer with Elecsys Anti-SARS-CoV-2 and Anti-SARS-CoV-2 S (Hitachi Ltd., Tokyo, Japan and Roche Diagnostics GmbH, Basel, Switzerland) kits. Samples exceeding the upper limit of detection were diluted and re-measured. Serum remainders were cryopreserved for analysis of neutralizing antibody titers (NAT). Subjects developing BTIs were excluded from the time of first positive nucleocapsid-specific IgG titer (>1.0 cut-off index). Likewise, subjects who missed a follow-up measurement were excluded from all subsequent timepoints due to unknown BTI status. NAT were determined as previously described (18).

### 2.3 Preparation of stimulation tubes for whole blood-based immunoassays

As published previously (19, 20), 2.7-mL blood collection tubes without anticoagulant were pre-loaded with co-stimulatory antibodies α-CD28 and α-CD49d plus the following stimuli: (i) negative control (no antigen), (ii) SARS-CoV-2 S peptides, (iii) or positive control (mixture of CPI and CEF peptides). Details regarding antigens, reagents, manufacturers, and concentrations are provided in **Supplementary Table S2**.

### 2.4 Flow cytometric characterization of spike-reactive T cells

Stimulation tubes prepared as described above were inoculated with 500 µL whole blood. After 4 h of incubation at 37°C, brefeldin A was added at a final concentration of 10 µg/mL, followed by incubation for another 16-18 h at 37°C. After erythrocyte lysis using Buffer EL (Qiagen, Hilden, Germany), extracellular staining was performed using CD197(CCR7)-BV650 (BioLegend, San Diego, USA), CD3-VioBright R720, CD4-VioBlue, CD8-VioGreen, CD45RO-APC-Vio770, CD137-APC, CD279(PD1)-VioBright 515, and 7-AAD (all from Miltenyi Biotec, Bergisch Gladbach, Germany) according to the manufacturer’s instructions. Intracellular staining with CD69-PE-Vio615, CD154-PE-Vio770, and IFN-γ-PE (all from Miltenyi Biotec) was performed using the Inside Stain Kit (Miltenyi Biotec) according to the manufacturer’s instructions. Stained cells were analyzed using a Cytoflex S flow cytometer (Beckman Coulter, Brea, USA) and Cytexpert software (version 2.4.0.28, Beckman Coulter, Brea, USA). Downstream analysis was performed with Kaluza (version 2.1, Beckman Coulter).

### 2.5 Functional characterization of spike-reactive T cells by cytokine and transcriptome analysis

For cytokine secretion assays and transcriptome analysis, stimulation tubes (**Supplementary Table S2**) were incubated for 20 – 24 h at 37°C without addition of brefeldin A. Concentrations of secreted cytokines were measured in supernatants using the 13-plex LegendPlex Essential Immune Response Kit (BioLegend).

The residual cell pellet underwent erythrocyte lysis and was then cryopreserved in 1 mL RNAprotect (Qiagen). Total RNA was isolated using the RNeasy Plus Mini Kit (Qiagen). Transcripts for 785 immune-related genes were quantified using the nCounter sprinter platform and Human Immune Exhaustion panel (Nanostring, Seattle, USA). Data was analyzed using the nSolver Analysis Software, with elimination of flagged datasets, background thresholding to the median of negative controls, and normalization to the panel’s 12 housekeeping genes (geometric mean). Thereafter, the ratio of normalized mRNA counts in S-stimulated samples versus unstimulated background controls was determined for each subject. After classification of subjects by serological and cytokine responses, as detailed in the Results section, median S-stimulated/background ratios were determined for each cohort. Median-to-median ratios (MMRs) between high- and low-responders were calculated and imported into Ingenuity Pathway Analysis (Qiagen). Core analysis was performed to determine canonical pathway enrichment. Enrichment of canonical pathways was considered significant at a Benjamini-Hochberg adjusted p-value <0.05. For inclusion in figures and to determine biological significance of pathway activation/inhibition, absolute z-score thresholds of ≥1.5 and ≥2 were applied, respectively. The data discussed in this publication have been deposited in NCBI’s Gene Expression Omnibus (21) and are accessible through GEO Series accession number GSE273994 (https://www.ncbi.nlm.nih.gov/geo/query/acc.cgi?acc=GSE273994).

### 2.6 Statistical analysis

To determine S-specific T cell frequencies and cytokine release, results of unstimulated controls were subtracted from S-stimulated responses. Unless indicated otherwise, individual results and medians are shown. Spearman rank coefficients were used for correlation analysis. Depending on the data format, Mann-Whitney-U-test, Wilcoxon’s rank sum test, Kruskal-Wallis test, or Friedman test were used for significance testing, as specified in the figure legends. Dunn’s post-test was used for multi-group comparisons. For analyses with 5 or more readouts, the Benjamini-Hochberg test was used to identify significant results with a false discovery rate of <0.2. Data analysis and visualization was performed using Excel 365 (Microsoft Corporation, Redmond, USA) and Prism v9 (GraphPad Software, Boston, USA).

Support Vector Machine (SVM) analyses were performed using an online calculator (22). Top and bottom responder classification of subjects at T6 (based on S IgG, S-induced IL-2, IFN-γ, or a combination of all three readouts) were predicted by training linear kernel models with independent variables pre-selected using the significance tests described above. Models were generated in multiple iterations by successively adding independent variables to those yielding the highest F1 scores in the previous iteration. This process was repeated until either all additional variables worsened F1 scores, no further improvement in F1 score was achieved in two successive iterations, a maximum of four markers had been selected, or an optimal result was achieved (F1 = 1.00). Among combinations of independent variables yielding the same F1 score, those with the fewest independent variables were selected.

## 3 Results

### 3.1 Multifaceted humoral and cellular anti-spike response peak at 1 month post-booster

We enrolled a total of 30 subjects at T0. Five of them were male and 25 were female. Their median age was 51 years (range 26-65 years). Only 7 subjects had a known previous SARS-CoV-2 infection. One subject at T1, 6 at T3, and 14 at T6 were excluded due to positive N antibodies or missed follow-up (**Supplementary Figure S1**).

**Figure 1:**
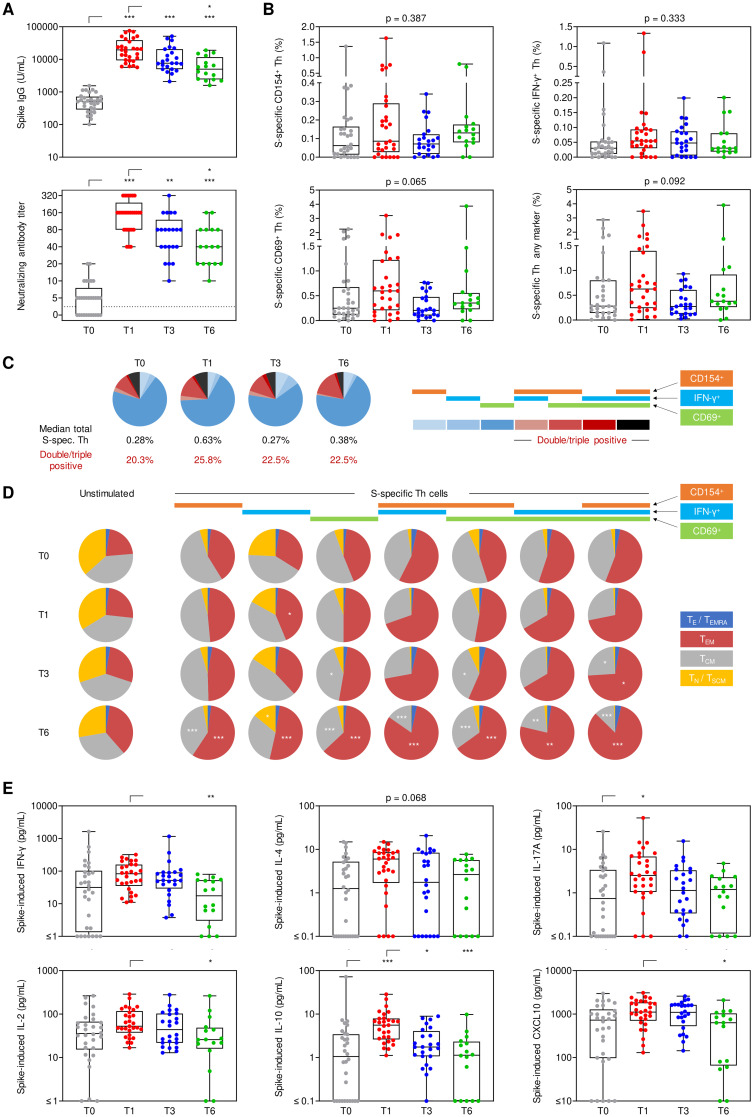
Longitudinal evolution of spike-specific immune responses. Humoral and cellular spike (S)-specific immune responses were monitored before booster vaccination (T0) as well as 1 (T1), 3 (T3), and 6 months (T6) post-booster. **(A)** S IgG and neutralizing antibody titers. **(B)** S-specific Th cell frequencies among viable CD3^+^CD4^+^ lymphocytes based on the expression of CD154, IFN-γ, and/or CD69. **(C)** Characterization of the (background-adjusted) S-specific Th cell population expressing one or more of the activation markers CD154 (orange), IFN-γ (blue) or CD69 (green). **(D)** Phenotypic characterization of unstimulated and S-reactive Th cells based on CD45RO and CCR7 expression levels: T_E_/T_EMRA_ (blue), CD45RO^-^CCR7^-^; T_EM_ (red), CD45RO^+^CCR7^-^; T_CM_ (grey), CD45RO^+^CCR7^+^; T_N_/T_SCM_ (yellow), CD45RO^-^CCR7^+^. **(E)** Background-adjusted S-induced secretion of IFN-γ, IL-2, IL-4, IL-10, IL-17A, and CXCL10. **(A, B, E)** Individual values, median, interquartile range (boxes), and spread (whiskers) are shown. Paired/matched analyses are shown in **Supplementary Figure S2** and **Supplementary Figure S3**. **(A–C, E)** Kruskal-Wallis test with Dunn’s post-test. * p<0.05, ** p<0.01, *** p<0.001. Significance indicators in **(C)** refer to comparisons at T1/T3/T6 *versus* T0. S, spike; T_E_/T_EMRA_, effector T cells and effector memory T cells re-expressing CD45RA; T_EM_, effector memory T cells; T_CM_, central memory T cells; T_N_/T_SCM_, naïve T cells and stem cell memory T cells.

Compared to baseline (pre-booster), we observed strong and significant increases in S IgG and NAT in all subjects 1 month after booster vaccination (median NAT of 160 at T1 vs. 5 at T0, p<0.001, **Figure 1A**), followed by a steady decrease during the 6-month follow-up period (median NAT of 80 and 40 at T3 and T6, respectively). These trends were corroborated by matched analysis of subjects with complete follow-up data (T0 vs. T1 in **Supplementary Figure S2A**, T0 vs. T1 vs. T6 in **Supplementary Figure S3A**).

Likewise, S-specific Th-cell reactivity, as measured by intracellular expression of CD154 (median, 0.086% at T1 vs. 0.062% at T0), CD69 (0.597% vs. 0.248%), and IFN-γ (0.054% vs. 0.030%), or any combination of these markers (0.627% vs. 0.282%), moderately increased after booster vaccination (**Figure 1B**; **Supplementary Figures S2B**, **S3B**). However, due to considerable inter-individual baseline variation, statistical significance for most markers was only seen upon paired T0/T1 analysis (CD154, p=0.014; IFN-γ, p<0.001; any marker, p=0.048, **Supplementary Figure S2B**).

At all timepoints, >20% of S-specific Th cells expressed more than one activation marker, with a minor and non-significant increase at T1 (25.8% double/triple-positive, **Figure 1C**). With double- and triple-positivity, the predominant phenotype of S-specific Th cells shifted from a central memory phenotype (T_CM_) to an effector memory phenotype (T_EM_) from T1 onwards (**Figure 1D**). Likewise, the proportion of S-specific T_EM_ Th cells increased while the T_CM_ phenotype decreased after booster vaccination compared to baseline, especially at T6 (p<0.01 for 7/7 [T_EM_] and 6/7 [T_CM_] combinations of activation markers, respectively, **Figure 1D**).

Notably, S-induced release of several T-cellular cytokines (IFN-γ, IL-2, IL-4, IL-10, IL-17) and chemokines associated with IFN-γ release (CXCL10) in S-stimulated whole blood was markedly stronger at T1 vs. T0 (**Figure 1E**), with statistical significance reached for all markers in at least one of the two paired/matched analyses (**Supplementary Figures S2C**, **S3C**). S-induced release of all cytokines reverted to pre-booster levels by T6 (**Figure 1E**, **Supplementary Figures S2C**, **S3C**). Taken together, these findings indicate broad yet mostly short-lived expansion and activation of S-specific Th cells, paralleled by a more persistent shift in effector/memory phenotypes of Th cells.

### 3.2 IFN-γ^+^ S-specific Th cells present pre-booster shape cytokine and antibody responses

For all post-booster assessments, we observed high inter-individual variability of serological and T-cell responses (**Figure 1**). Most readouts showed no association with age. However, increasing age correlated with higher S IgG at T1 (ρ=0.52, p=0.004) but less robust sustained S-specific T-cell responses at T6 (significant for % S-specific Th cells, ρ=-0.60, p=0.016) (**Supplementary Figure S4**).

**Figure 2:**
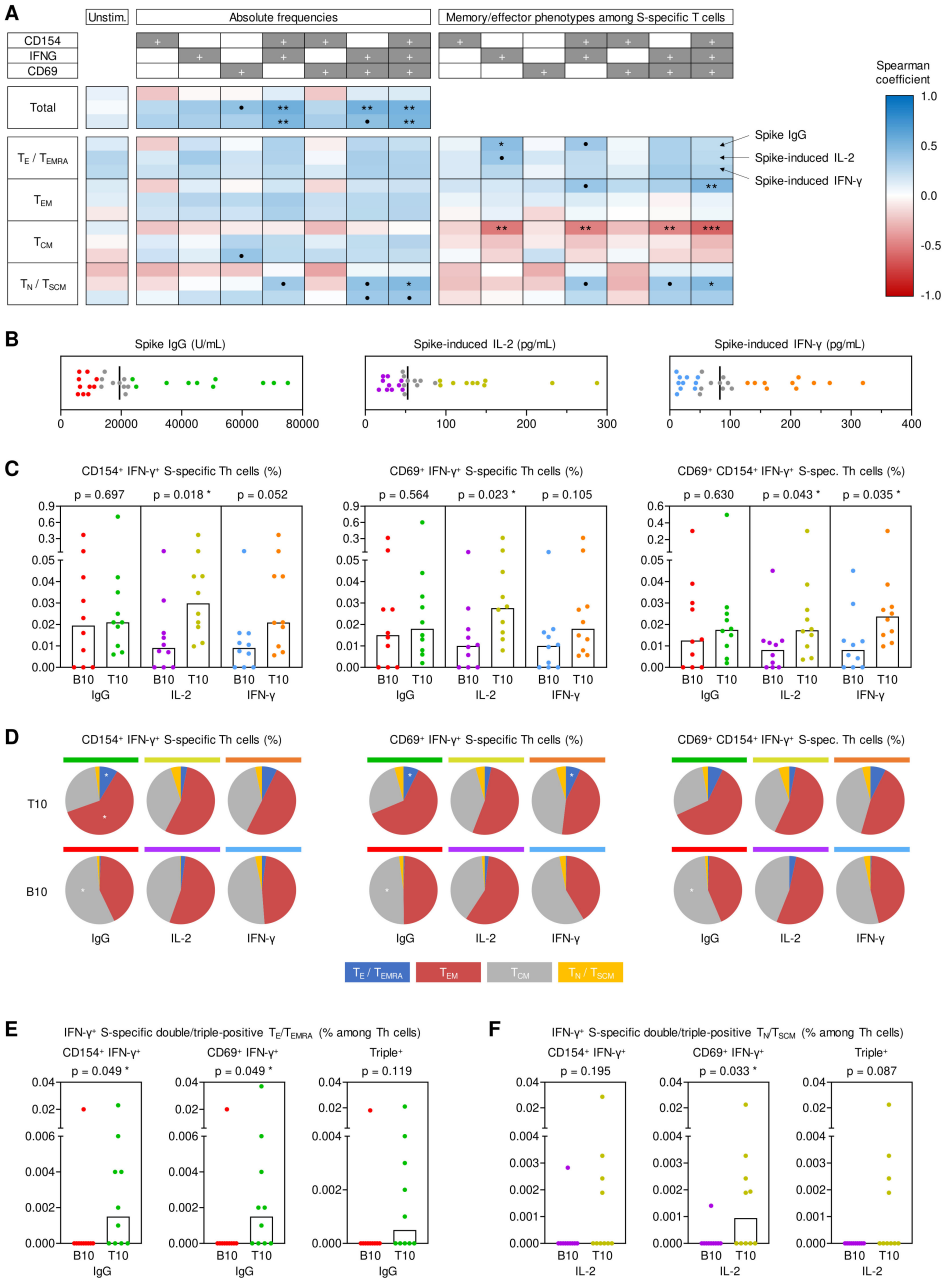
Impact of antigen-specific Th cells at T0 on key determinants of anti-spike immunity at T1. **(A)** Spearman correlation of pre-existing (T0) spike (S)-specific T helper (Th) cells (expressing CD154, IFN-γ, and/or CD69) and their respective memory subsets (T_E_/T_EMRA_, CD45RO^-^CCR7^-^; T_EM_, CD45RO^+^CCR7^-^; T_CM_, CD45RO^+^CCR7^+^; T_N_/T_SCM_, CD45RO^-^CCR7^+^) with T1 S IgG, S-induced IL-2 release and S-induced IFN-γ release. **(B)** Determination of bottom-10 (B10) and top-10 (T10) responders based on T1 serum S IgG levels (red/green), S-induced IL-2 secretion (purple/gold), and S-induced IFN-γ secretion (blue/orange). Median values are indicated by black bars. **(C)** T0 frequencies of double- and triple-positive IFN-γ^+^ S-specific Th cells among Th lymphocytes depending on response classification at T1. **(D)** Phenotypic characterization of IFN-γ^+^ double- and triple-positive S-specific Th cells at T0 depending on response classification at T1: T_E_/T_EMRA_ (blue), T_EM_ (red), T_CM_ (grey), T_N_/T_SCM_ (yellow). **(E)** Frequencies of pre-existing IFN-γ^+^ S-specific double- and triple-positive T_E_/T_EMRA_ among Th cells in B10 and T10 S IgG producers at T1. **(F)** Frequencies of pre-existing IFN-γ^+^ S-specific double- and triple-positive T_N_/T_SCM_ among Th cells in B10 and T10 IL-2 responders to S stimulation at T1. **(C, E, F)** Individual and median values (columns) are shown. Mann-Whitney-U test. • p<0.05, false discovery rate (FDR)>0.2; * p<0.05, FDR<0.2; ** p<0.01, FDR<0.2; *** p<0.001, FDR<0.2. B10, T10, bottom 10, top 10 responder; FDR, false discovery rate; S, spike; T0, T1, T6, 0, 1, or 6 months post booster; Th, T helper cells; T_E_/T_EMRA_, effector T cells and effector memory T cells re-expressing CD45RA; T_EM_, effector memory T cells; T_CM_, central memory T cells; T_N_/T_SCM_, naïve T cells and stem cell memory T cells.

We hypothesized that inter-individual variation may be caused by differences in memory and effector Th subsets present at the time of booster vaccination (i.e., at T0). Consistent with a previous study (23), we found no significant correlation between NAT and S-specific T cells in our cohort. Therefore, we focused on correlations of frequencies of S-specific T cells, their phenotypes, and double- or triple-activation marker positivity at T0 with post-booster S IgG as well as S-specific IFN-γ and IL-2 secretion, which are widely considered surrogates of protective adaptive immunity (24–26). Interestingly, we found a strong and significant positive correlation of post-booster (T1) cytokine responses with total pre-booster double- and triple-positive IFN-γ producing S-specific Th cells (ρ=0.45-0.56), especially the naive/stem cell memory (T_N_/T_SCM_) sub-population (ρ=0.26-0.48, **Figure 2A**). Additionally, we found a strong association of S-specific IFN-γ or IL-2 release at T1 with S-specific IL-2 at T0 (ρ=0.62-0.77, p<0.001), a cytokine known to be abundantly produced by T_SCM_ cells (27, 28) (**Supplementary Figure S5A**).

Although S IgG serum levels at T1 did not correlate with absolute pre-booster frequencies of S-specific T cells, we found a modest positive correlation of S IgG levels at T1 with differentiated (T_E_/T_EMRA_) IFN-γ^+^ S-specific T cells and a higher proportion of T_E_/T_EMRA_ or T_EM_ IFN-γ^+^ S-specific T cells at T0 (**Figure 2A**). Moreover, S IgG levels at T1 showed a significant positive correlation (ρ=0.39-0.54) with S-induced release of various Th1, Th17, and associated innate effector cytokines at T0 (**Supplementary Figure S5A**). Collectively, these results indicate significant contributions of antigen-specific Th cells present pre-booster, especially IFN-γ^+^ (Th1) cells, to antibody and cellular responses after booster vaccination. Moreover, these findings suggest that distinct baseline phenotypes of pre-existing antigen-specific Th cells are associated with antibody (T_E_/T_EMRA_) versus T-cellular cytokine responses (T_N_/T_SCM_).

To corroborate these results, we used a previously described (8) “top/bottom-10” approach to classify antibody and T cellular responses at T1 (**Figure 2B**). Based on this classification, pre-existing S-specific IFN-γ^+^ double- and triple-positive Th frequencies were significantly associated with S-induced IL-2 release at T1 (p=0.018-0.043). Likewise, triple-positive (IFN-γ^+^CD69^+^CD154^+^) S-specific T cell frequencies at T0 were significantly associated with S-induced IFN-γ release at T1 (p=0.035, **Figure 2C**). Although top-10 and bottom-10 S IgG responders showed similar frequencies of total double- and triple-positive IFN-γ producing Th cells (**Figure 2C**), top-10 S IgG responders had increased proportions of pre-existing S-specific double- and triple-positive IFN-γ producing T_EM_ and T_E_/T_EMRA_ cells at T0 (**Figure 2D**), consistent with the correlation analysis shown above. In fact, only one of the bottom-10 S IgG responders at T1 had measurable quantities of these cells at T0, whereas most top-10 responders had S-specific IFN-γ producing T_EM_ and T_E_/T_EMRA_ cells co-expressing at least one of the activation markers CD69 and/or CD154 (p=0.049, **Figure 2E**). *Vice versa*, compared to bottom-10 responders, top IL-2 responders at T1 had more IFN-γ^+^ S-specific double- and triple-positive T_N_/T_SCM_ cells at T0, especially IFN-γ^+^ CD69^+^ cells (p=0.033, **Figure 2F**). Additionally, the top/bottom-10 classification also corroborated significantly greater S-induced production of IL-2 (p=0.019) and IL17-A (p=0.014) at T0 in top-10 T1 spike IgG producers, with similar yet non-significant trends for IFN-γ (p=0.061), MCP1 (p=0.073), and TNF-α (p=0.052) (**Supplementary Figure S5B**).

Altogether, these analyses suggest that S-reactive double- and triple-positive IFN-γ^+^ Th cells present before booster vaccination are strongly associated with short-term serological and T-cellular post-booster responses, with disparate baseline phenotypes favoring stronger T-cell versus serological responses. Specifically, avid T-cellular cytokine responses seem to rely on pre-existing double- and triple-positive IFN-γ T cells, including less-differentiated populations (T_N_/T_SCM_), while S-specific T_EM_ and T_E_/T_EMRA_ cells present pre-booster contributed to robust S-specific IgG levels.

### 3.3 Cytokine production of S-specific Th cells at 1 month post-booster determines sustained reactivity of the cellular and humoral immune response at 6 months post-booster

**Figure 3:**
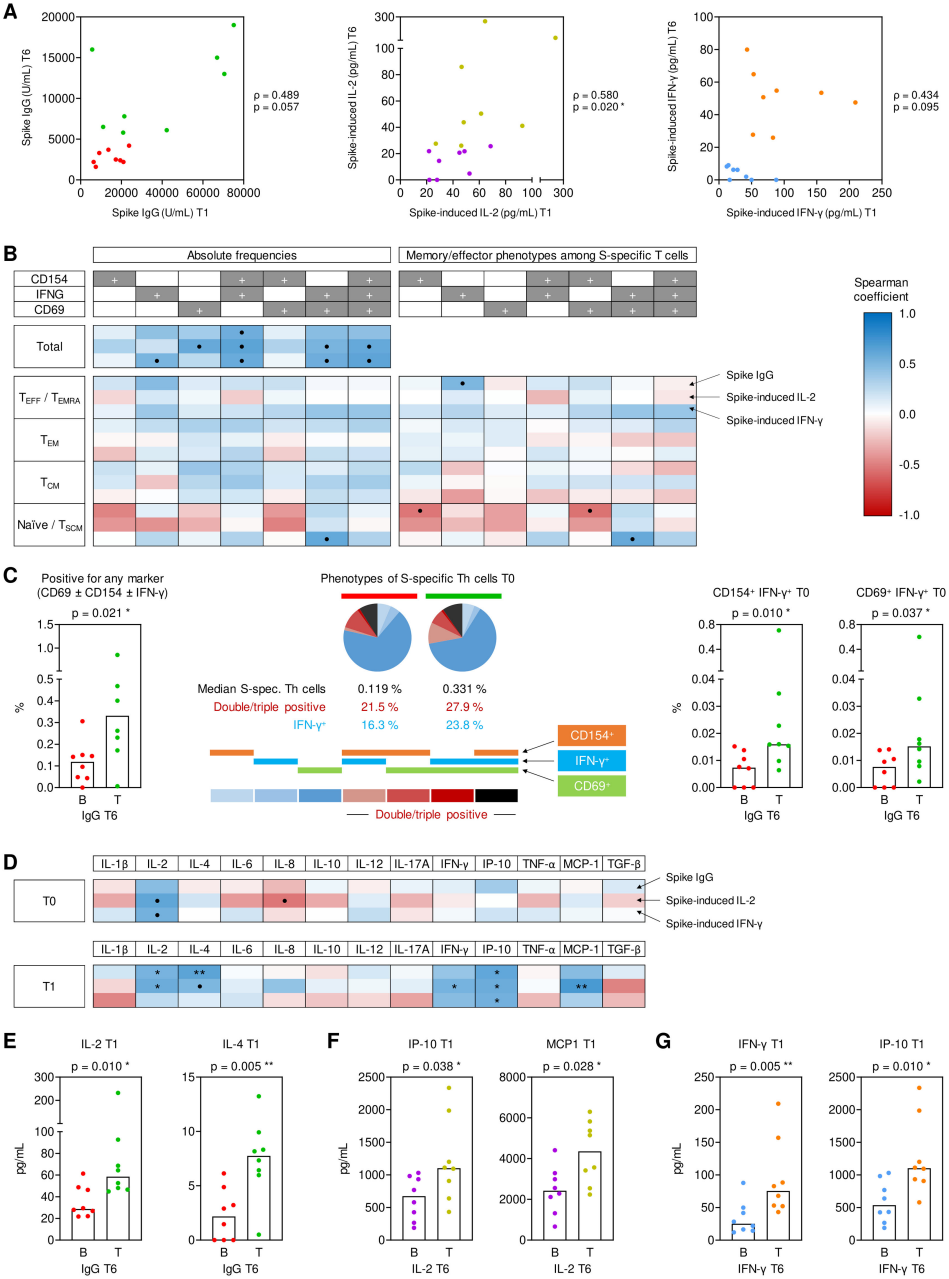
Pre- and post-booster predictors of sustained antibody and cytokine responses at T6. **(A)** Spearman correlation of T1 (peak) and T6 (long term) spike (S)-specific IgG levels, S-induced IL-2 release, and S-induced IFN-γ release. Bottom-half **(B)** and top-half (T) long-term responses are color-coded in red/green for IgG levels, purple/gold for S-induced IL-2, and blue/orange for S-induced IFN-γ based on T6 values. **(B)** Spearman correlation of S-specific Th cells (expressing CD154, IFN-γ, and/or CD69) and their respective effective/memory subsets (T_E_/T_EMRA_, CD45RO^-^CCR7^-^; T_EM_, CD45RO^+^CCR7^-^; T_CM_, CD45RO^+^CCR7^+^; T_N_/T_SCM_, CD45RO^-^CCR7^+^) at T1 with measurements of S IgG, S-induced IL-2, and S-induced IFN-γ at T6. **(C)** Frequencies of pre-existing (T0) IFN-γ^+^ S-specific Th cells of B (red) and T (green) S IgG producers at T6 and characterization based on the expression of one or more activation markers (CD154, IFN-γ, and/or CD69). **(D)** Spearman correlation of S-induced cytokine secretion levels at T0 (upper panels) and T1 (lower panel), respectively, with S IgG, S-induced IL-2, and S-induced IFN-γ at T6. **(E)** S-induced IL-2 and IL-4 secretion at T1 of bottom- (red) and top-half (green) long term S IgG producers at T6. **(F)** S-induced IP-10 and MCP1 secretion at T1 of bottom- (purple) and top-half (gold) long term IL-2 responders to S stimulation at T6. **(G)** S-induced IFN-γ and IP-10 secretion at T1 of bottom- (blue) and top-half (orange) long-term IFN-γ responders to S stimulation at T6. **(C, E-G)** Individual and median values (columns) are shown. Mann-Whitney-U test. • p<0.05, false discovery rate (FDR)>0.2; * p<0.05, FDR<0.2; ** p<0.01, FDR<0.2; *** p<0.001, FDR<0.2. B, T, bottom, top half long term responder; FDR, false discovery rate; S, spike; T0, T1, T6, 0, 1, or 6 months post booster; Th, T helper cells; T_E_/T_EMRA_, effector T cells and effector memory T cells re-expressing CD45RA; T_EM_, effector memory T cells; T_CM_, central memory T cells; T_N_/T_SCM_, naïve T cells and stem cell memory T cells.

Given the marked inter-individual variation in long-term serological and T cell responses (**Figures 1A, B**), we next sought to elucidate whether antigen-specific Th cells present pre-booster (T0) and/or the early booster-induced response determine S-specific adaptive immunity at T6. S IgG levels and S-induced release of IL-2 and IFN-γ at T1 were found to moderately correlate with their respective levels at T6 (ρ=0.43-0.58, **Figure 3A**), suggesting partially overlapping immunological determinants of short- and long-term post-booster responses.

Double- and triple-positive IFN-γ^+^ S-specific Th cells at T0 strongly correlated with both S IgG levels and S-induced release of IL-2 and IFN-γ at T6 (ρ=0.54-0.64), whereas no consistent association with specific effector/memory Th phenotypes was seen (**Figure 3B**). Accordingly, the total pre-booster frequencies of S-specific T cells, the proportion of IFN-γ^+^ S-specific T cells, the proportion of double- and triple-positive S-specific T cells, and frequencies of IFN-γ^+^ S-specific T cells co-expressing either CD69 or CD154 were significantly higher in the top half of serological responders at T6 than in bottom-half responders (p=0.010-0.037, **Figure 3C**).

In sharp contrast, not S-specific T-cell frequencies (**Supplementary Figure S6**) but Th cytokine release and associated innate effector cytokines at T1 (**Figure 3D**) were significantly associated with S IgG levels and S-induced release of IL-2 and IFN-γ at T6. Specifically, S-induced release of IL-2, the Th1 signature cytokine IFN-γ, the IFN-γ-induced effectors IP-10 and MCP1, and the Th2 signature cytokine IL-4 at T1 were significantly associated with at least one of the three studied surrogates of long-term (T6) responses (**Figures 3D-G**).

Altogether, these data support the following model: Short-term antibody responses are mainly determined by pre-existing IFN-γ^+^ S-specific Th cells. Early post-booster T cell activation, Th cytokine release, and associated cytokine responses are determined by total double- and triple-positive S-specific Th-cell frequencies, including less differentiated phenotypes (T_N_/T_SCM_). Early post-booster T-cell activation and cytokine release, in turn, promotes long-term S-specific antibody and T cellular immunity.

### 3.4 Transcriptomic analysis reveals upregulation of both adaptive and innate immunity pathways in top responders

**Figure 4:**
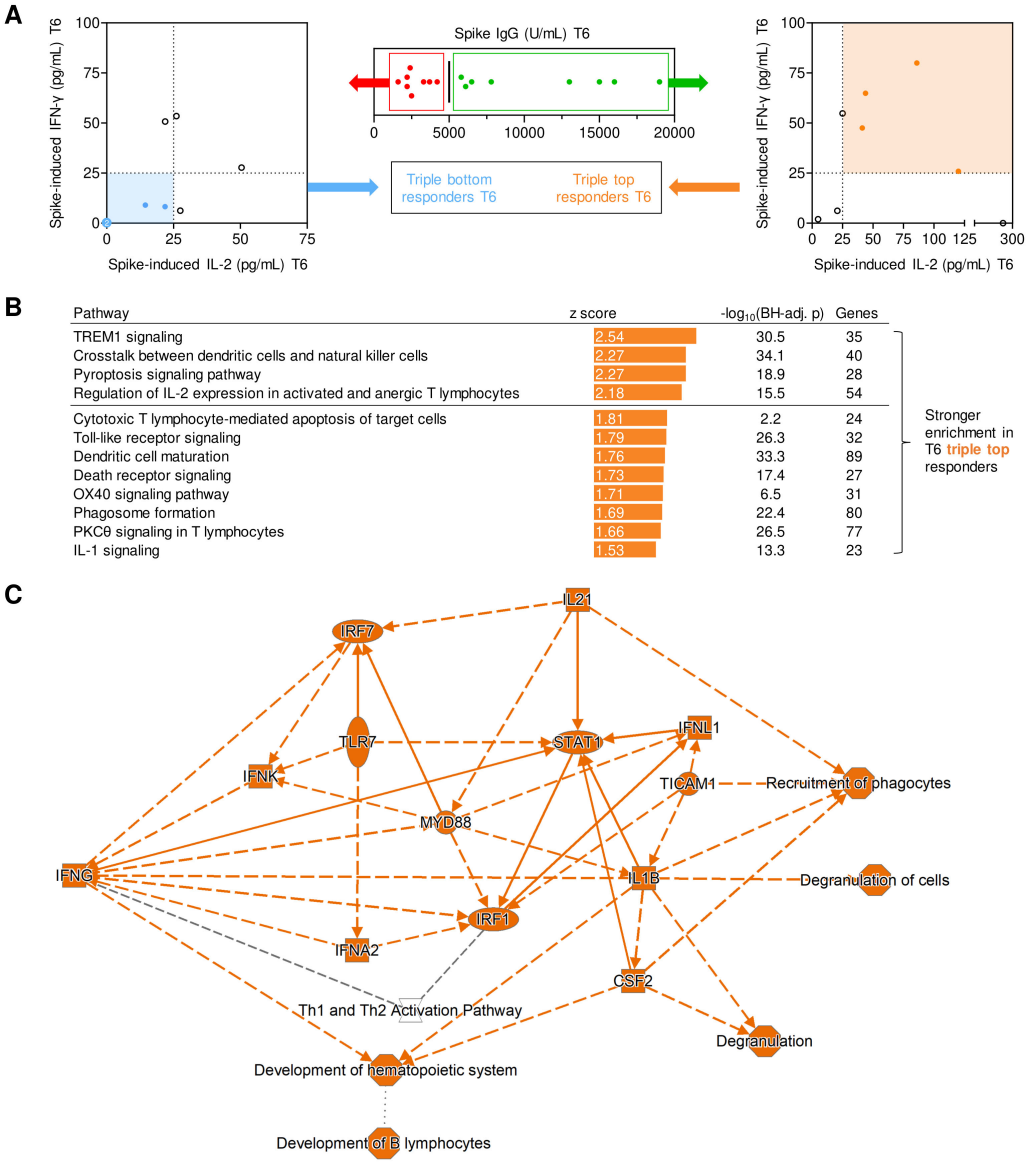
Transcriptional networks associated with long-term post-booster responses. **(A)** Screening algorithm used to identify triple top- and bottom-responders at T6 based on spike (S)-specific IgG, S-induced IL-2 release, and S-induced IFN-γ release. **(B)** Pathways with significantly stronger expression at T1 in T6 triple-top responders. Pathways with absolute z-scores ≥1.5 and Benjamini-Hochberg-adjusted (BH-adj.) p-values <0.05, i.e., -log_10_(BH-adj. p) ≥1.3 are displayed. No pathways suppressed in triple-top responders reached the z ≤-1.5 threshold. Activated pathways with absolute z-scores ≥2 are considered biologically significant (horizontal line). **(C)** (Simplified) gene expression network and predicted interactions, as determined by Ingenuity Pathway Analysis (core analysis, graphical summary). Orange interaction nodes indicate stronger induction/activation at T1 in T6 triple-top responders. BH-adj., Benjamini-Hochberg-adjusted; S, spike; T1, T6, 1 or 6 months post booster; Th, T helper cells.

To obtain a more detailed picture of the early post-booster immune landscape associated with long-term serological and T cellular responses, we performed n-Counter-based transcriptomics and pathway enrichment analysis on S-stimulated whole blood at T1 (**Figure 4A**) and compared individuals classified as T1 and T6 triple (S IgG, IL-2, and IFN-γ) top versus bottom responders (**Figure 4B**). To functionally validate transcriptional analyses from stimulated whole blood, we first compared T1 pathway enrichment based on T1 response classification. Indeed, we found strong and highly significant positive correlations between T1 cytokine responses and expression of a broad array of adaptive and innate effector pathways, whereas exhaustion pathways (CTLA-4 and PD-1) were suppressed in triple top responders (**Supplementary Figure S7**).

When subsequently comparing transcriptional pathway enrichment at T1 based on their response classification at T6, individuals with high levels of S IgG, S-induced IL-2, and IFN-γ at T6 displayed enrichment of several pathways associated with strong adaptive immune activation (e.g., IL-2 signaling, OX40 signaling) at T1 (**Figure 4C**). Interestingly, pathway analysis at T1 also revealed that several pathways and genes involved in pattern recognition receptor signaling, innate immune cell activation, antigen presentation, and inter-cellular crosstalk were significantly stronger activated in T6 IgG, IL-2, and IFN-γ top responders than in bottom responders (**Figure 4C**). In summary, these findings support a mechanistic link between short- and long-term immune protection and suggest that feedback loops between pre-existing and/or newly formed S-specific T cells and antigen-presenting cells (APCs) might contribute to both short- and long-term adaptive immune responses.

### 3.5 S-specific Th-cell present at the time of booster vaccination and surrogates of early post-booster responses predict sustained production of spike-specific IgG, IL-2, and IFN-γ

Given our prior observations that both antigen-specific Th-cell frequencies pre-booster and early post-booster responses were associated with surrogates of adaptive long-term immune responses, we next analyzed the predictive performance of such markers for stratification of individual long-term (T6) responses. To that end, we tested various combinations of markers that significantly correlated with S IgG and S-specific secretion of IL-2 and IFN-γ at T6 (**Figures 3E, F**) and performed SVM analyses.

Here, a combination of pre-existing S-specific Th cells or, specifically, IFN-γ^+^ double- and triple-positive Th cells at T0 and S IgG, S-induced release of IFN-γ, IL-2, IL-4, IP-10, and MCP-1 at T1 predicted top- versus bottom-half classification of S IgG and S-specific secretion of IL-2 and IFN-γ with high precision and recall performance (≥0.80 and 1.00, respectively), leading to F1 scores of ≥0.89. Further, the above combination of pre-booster and early post-booster markers showed 100% precision, recall, and accuracy for identification of triple (i.e., S IgG and S-induced IFN-γ and IL-2) top and bottom responders at T6 (**Table 1A**). Remarkably, inclusion of T1 S IgG as an immune variable consistently led to inferior predictive F1 scores (**Supplementary Figure S8**). Altogether, these findings underscore the importance of functional T-cell analyses in the monitoring of adaptive immunity after SARS-CoV-2 booster vaccinations and corroborate a model whereby S-specific Th cells detectable pre-booster and the magnitude and quality of early post-booster Th-cell jointly determine long-term adaptive immune protection.

**Table 1:** Support vector machine analysis of T0 and T1 immune markers to predict sustained spike-specific (S) IgG and S-induced cytokine response at T6.

T6 outcome	Combination yielding highest F1 score		F1 score	Precision	Recall	Accuracy
(A) Including flow cytometric readouts						
S IgG	T1	IL-2	0.89	0.80	1.00	0.88
	T1	IP-10				
S-specific IL-2	T0	S-specific Th cells	0.94	0.89	1.00	0.94
	T1	IL-4				
	T1	IP-10				
	T1	MCP-1				
S-specific IFN-γ	T0	IFN-γ^+^ MF Th cells	1.00	1.00	1.00	1.00
	T1	IFN-γ				
	T1	IL-2				
	T1	IP-10				
Triple classification	T0/1	8 possible combinations of 2 markers each ^a^	1.00	1.00	1.00	1.00
(B) Cytokine release only						
S IgG	T1	IL-2	0.89	0.80	1.00	0.88
	T1	IP-10				
S-specific IL-2	T1	MCP-1	0.80	0.86	0.75	0.81
S-specific IFN-γ	T1	IFN-γ	0.94	0.89	1.00	0.94
	T1	IP-10				
Triple classification	T1	3 possible combinations of 2 markers each ^b^	1.00	1.00	1.00	1.00

a (i) T0 IFN-γ^+^ MF Th cells + T1 IFN-γ, (ii) T0 IFN-γ^+^ MF Th cells + T1 IL-2, (iii) T0 IFN-γ^+^ MF Th cells + IL-4, (iv) T0 IFN-γ^+^ MF Th cells + T1 MCP-1, (v) T0 S-specific Th cells + T1 IP-10, (vi) T1 IFN-γ + T1 IP-10, (vii) T1 IFN-γ + T1 MCP-1, or (viii) T1 IP-10 + T1 MCP-1.

b (i) IFN-γ + IP-10; (ii) IFN-γ + MCP-1, and (iii) IP-10 + MCP-1.

MF, multifunctional (i.e., double-/triple-positive); S, spike; T0, T1, T6, timepoints 0, 1, or 6 months post booster-vaccination; Th, T helper cells.

However, due to the limited feasibility of flow cytometric analysis for clinical routine use (29–33), and to avoid reliance on repeat sampling at T0 and T1, we compiled additional SVM models relying exclusively on S-induced cytokine release at T1 (**Supplementary Figure S9**, **Table 1B**). Although some of the resulting F1 scores were slightly lower than in the initial model including flow cytometry at T0, classification of long-term responses based on S-induced cytokine release at T1 was feasible with high F1 scores of 0.80-1.00 (**Supplementary Figure S9**, **Table 1B**). In particular, cytokine release assays at T1 facilitated correct prediction of all triple top and bottom responders at T6.

## 4 Discussion

Several groups have characterized the cellular adaptive immune response to primary SARS-CoV-2 vaccination, whereas the cellular determinants of sustained immune protection after booster vaccinations are incompletely understood. Herein, we identified several components of antigen-specific Th-cell responses pre- and post-booster that are associated with strong and sustained serological and cytokine responses after booster vaccination. Specifically, peak S-specific IgG correlated with pre-existing IFN-γ^+^ T_E_/T_EMRA_ cells while double- and triple-positive IFN-γ^+^ Th cells present before booster vaccination, including T_N_/T_SCM_ subsets, were associated with stronger cytokine secretions in response to booster vaccination (especially IL-2 and IFN-γ). These cytokine responses in turn determined the long-term production of S IgG over the 6-month observation period. On the transcriptional level, IL-2, OX40, and other genes associated with T-cell proliferation, were indicative of sustained S-reactive cytokine release and stable S-specific IgG levels.

This underlines the cellular immune response’s essential role as a facilitator and predictor of antibody production and long-term adaptive immune protection after vaccinations. Specifically, interaction of T-cellular CD154 with its receptor CD40 on B cells is essential for germinal center formation, antibody class switching, and affinity maturation (34). Although we found that IFN-γ-producing effector and effector memory Th cells correlate with development of IgG responses, we cannot precisely pinpoint the causative T-cell type. The most obvious candidates would be T-follicular helper cells, which promote development of high-affinity IgG in B-cell follicle germinal centers (35). Previous studies showed concomitant development and comparable peak frequencies of circulating T-follicular helper and IFN-γ-producing Th cells following SARS-CoV-2 vaccination (36). Surprisingly, these T-cell populations both correlate with development of specific IgG responses (36). While it is counter-intuitive that IFN-γ-producing T-cells, in particular Th1 cells, might support IgG development, previous data indicate that type-1 T-follicular helper cells, which transiently produce IFN-γ, induce T-bet expression in B-cells which is required for development of antiviral IgG isotypes (37, 38). Alternatively, the association between IFN-γ and IgG production might reflect the concomitant development of T-follicular helper cells and IFN-γ-producing Th cells. Further studies using more defined Th-cell markers are needed to fully elucidate which cell types support the development of SARS-CoV-2-specific IgG, and the underlying mechanisms.

Furthermore, it was previously shown that S-specific CD4^+^ T_EM_ cells one month after the 2^nd^ dose of primary SARS-CoV-2 mRNA vaccination determined sustained responsiveness of S-specific T cell responses 6 months post-booster (39). Although not considered the driver of responses to primary SARS-CoV-2 vaccination, there has been evidence of pleiotropic effects of pre-existing cellular immunity on response to both primary and booster vaccinations. For instance, pre-existing memory B cells were associated with increased S-specific and neutralizing antibody levels (39). As discussed in the Introduction, pre-existing SARS-CoV-2-crossreactive T cells and their age-dependent phenotypes influenced the quality, avidity, and diversity of the developing adaptive responses to SARS-CoV-2 after the primary vaccination series (8). Herein, we found that responses to subsequent booster vaccinations tend to benefit from the S-specific (effector) memory cell pool developed in response to the initial vaccination.

Interestingly, our findings further suggest that feedback loops of antigen-specific T cells and APCs are critical for enhanced adaptive immune responses after booster vaccination. This is consistent with prior evidence suggesting that upregulated IFN-α and IFN-γ receptor genes, natural killer cell signaling pathways, and production of reactive oxygen species and nitric oxide in macrophages at day 3 after flu vaccination predicted increased inhibitory antibody levels against influenza hemagglutinin at day 28 (40).

Our findings might have translational implications. Given the natural inter-individual heterogeneity and variability of responses to mRNA vaccination, even in healthy and seemingly immunocompetent individuals (13, 15, 16), simple functional immunoassays might allow for individualized prediction of the quality or longevity of immune responses and guide recommendations regarding additional booster doses. By monitoring pre-existing spike-specific Th cells and early post-booster Th responses with simple whole blood-based assays, we obtained a robust prediction of the vaccinees’ anti-spike immune response at 6-months post-booster. Notably, in the SVM model presented here, evaluation based on T cellular and cytokine secretion data exceeded the predictive performance of early serological responses (i.e., spike IgG) alone.

This study has several limitations. Due to its small scale, we could not assess protection against severe or symptomatic SARS-CoV-2 BTIs. Nine of our 30 subjects developed anti-nucleocapsid antibodies indicative of BTIs but our study was not sufficiently powered to determine associations of the identified immune surrogates and BTI status. However, to ensure that we specifically analyze responses to the first COVID-19 booster vaccination after prior primary (2-dose) BNT162b2 vaccination, we excluded subjects developing anti-nucleocapsid antibodies indicative of a SARS-CoV-2 infection from subsequent immunoassay timepoints. These included subjects missing one or more appointments, as we could not rule out an intermittent infection due to the short half-life of nucleocapsid antibodies. This finally resulted in only 16 subjects available for the 6-month assessment, limiting statistical power. Additionally, we recruited our subjects from a limited pool of healthy and mostly middle-aged healthcare workers. Although some associations with the subjects’ age were seen, this study was not designed or powered to investigate nuanced age-dependent differences in T-cell biology and transcriptional profiles, as previously suggested (8, 41). Furthermore, we did not evaluate T-cell reactivity to mutated spike peptides of any variant of concern, where reduced vaccine efficacy might be expected (39, 42–44). Lastly, given possible underreporting of asymptomatic or mild infections and unreliability of N antibodies to trace infections in the distant past, we could not assess the role of prior COVID-19 infection on immune status and response to booster vaccination.

Despite these limitations, we identified T-cellular activation and cytokine markers predictive of short- and long-term response to mRNA booster immunization against SARS-CoV-2. We found that pre-existing S-specific Th cell populations and their respective cytokine profiles determine the quality and longevity of adaptive immune responses to booster vaccinations, underscoring the influence of pre-existing antigen-specific CD4^+^ T cells on the robustness of adaptive humoral responses. Given the rapid evolution of vaccine technologies, implementation of simple and facile whole blood-based T-cellular cytokine release assays in early stages of future vaccine trials could help to preemptively identify potential low responders. As our immunoassay platform could be adapted to any protein antigen, expansion of this technology to other vaccine-preventable diseases or even post-exposure prophylaxis would be easily feasible.

## Data Availability

The data presented in the study are deposited in NCBI’s Gene Expression Omnibus repository (21), accession number GSE273994 (https://www.ncbi.nlm.nih.gov/geo/query/acc.cgi?acc=GSE273994).
