# Supplementary material for: Antigen-specific T helper cells and cytokine profiles predict intensity and longevity of cellular and humoral responses to SARS-CoV-2 booster vaccination
Source: Front Immunol. 2024 Aug 29;15:1423766. doi: 10.3389/fimmu.2024.1423766 (PMC11390417; doi:10.3389/fimmu.2024.1423766)
Supplement: Supplementary file 1 [file DataSheet1.docx]

Antigen-specific T helper cells and cytokine profiles predict intensity and longevity of cellular and humoral responses to SARS-CoV-2 booster vaccination

***Supplementary Material***

**
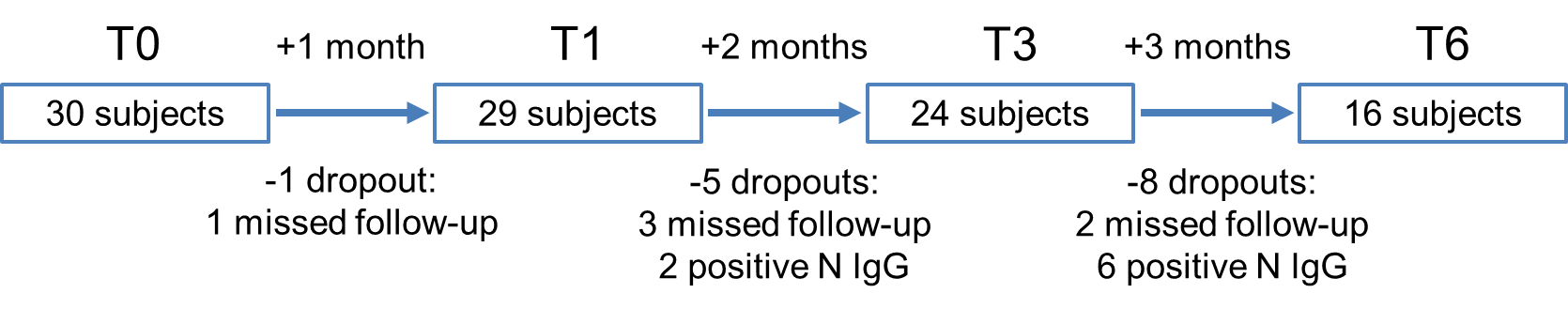
**

**Suppl. Figure S1. Schematic of study timeline including numbers of evaluable subjects per collection timepoint.**

Immune sampling was performed before (T0), 1 (T1), 3 (T3), and 6 months after (T6) booster vaccination. Reasons for study dropout were missed follow-up appointments (n=5) or positive serum nucleocapsid (N)-specific IgG (n=9). Abbreviations: N: nucleocapsid; T0, T1, T3, T6: 0, 1, 3, or 6 months post booster.

**Suppl. Figure S2. Pairwise analysis of humoral and cellular spike (S)-specific immune responses before (T0) and 1 month after (T1) booster vaccination.**

(**A**) S IgG and neutralizing antibody titers. (**B**) S-specific Th cell frequencies among viable CD3^+^CD4^+^ lymphocytes based on the expression of either CD154, IFN-γ, or CD69. (**C**) Background-adjusted S-induced secretion of IFN-γ, IL-2, IL-4, IL-10, IL-17A, and CXCL10. (**A**-**C**) N = 29 patients with T1 data. Neutralizing antibody titer was unavailable for one patient at T0 (n = 28). Paired Wilcoxon test. * p<0.05, ** p<0.01, *** p<0.001.

**Suppl. Figure S3. Matched analysis of humoral and cellular spike (S)-specific immune responses before (T0), 1 month after (T1), and 6 months after (T6) booster vaccination.**

(**A**) S IgG and neutralizing antibody titers. (**B**) S-specific Th cell frequencies among viable CD3^+^CD4^+^ lymphocytes based on the expression of either CD154, IFN-γ, or CD69. (**C**) Background-adjusted S-induced secretion of IFN-γ, IL-2, IL-4, IL-10, IL-17A, and CXCL10. (**A**-**C**) N = 16 patients with complete follow-up data. Friedman test with Dunn’s post test. * p<0.05, ** p<0.01, *** p<0.001. For IL-2 in panel C, the Friedman test yielded a significant p-value for comparison of all 3 timepoints; however, none of the pairwise comparisons reached multiple testing-adjusted significance.

**Suppl. Figure S4. Correlation of age at booster vaccination with spike (S)-specific immune responses at T1 and T6.**

Correlation analysis of age at booster vaccination with S IgG, S-specific Th cells (any combination of CD154^+^, CD69^+^, and/or IFN-γ^+^), and background-adjusted S-induced IFN-γ and IL-2 release at 1 (T1, n=29) and 6 (T6, n=16) months after booster vaccination. ρ = Spearman’s rank correlation coefficient. * p<0.05; ** p<0.01. Abbreviations: S: spike; T1, T6: 1 or 6 months post booster; U: units.

**Suppl. Figure S5. Association of spike (S)-induced cytokine release at T0 and T1 with S-specific IgG as well as S-induced IFN-γ and IL-2 release at T1.**

(**A**) Spearman correlation of S-induced cytokine release at T0 and T1 with measurements of S IgG, S-induced IL-2, and S-induced IFN-γ at T1. (**B**) Comparison of S-induced cytokine release at T0 in top-half (T) and bottom-half (B) S IgG producers at T1. Columns indicate medians. * p<0.05, false discovery rate (FDR)<0.2; ** p<0.01, FDR<0.2; *** p<0.001, FDR<0.2. Abbreviations: B, T: bottom-, top-half responders; FDR: false discovery rate; S: spike; T0, T1: 0, or 1 month post booster.

**Suppl. Figure S6. Correlation of spike- (S) specific T-helper cells at T1 and long-term adaptive immune responses against at T6.**

Correlation matrix of absolute (denominator, viable CD3^+^CD4^+^ cells) and relative (denominator, activation-marker-positive CD3^+^CD4^+^ cells) S-specific T-helper cell frequencies at T1 with S IgG and background-adjusted S-induced cytokine release at T6. ● p<0.05, false discovery rate (FDR)>0.2 (no results with FDR <0.2). Abbreviations: S: spike; T1, T6: 1 or 6 months post booster; T_EFF_/T_EMRA_: effector T cells and effector memory T cells re-expressing CD45RA; T_EM_: effector memory T cells; T_CM_: central memory T cells; Naïve/T_SCM_: naïve T cells and stem cell memory T cells.

**Suppl. Figure S7. Association of gene expression networks and phenotypic adaptive cellular and humoral anti-spike (S) responses at T1.**

(**A**) Screening matrix used to identify triple top- and bottom-responders at T1 based on spike-specific (S) IgG, S-induced IL-2 secretion, and S-induced IFN-γ secretion. (**B**) Pathways expressed significantly higher in either triple-top or triple-bottom responders. Pathways with absolute z-scores ≥1.5 and Benjamini-Hochberg-adjusted (BH-adj.) p-values <0.05, i.e., -log_10_(BH-adj. p) ≥1.3 are displayed. Pathways with absolute z-scores ≥2.0 are considered biologically significant (horizontal line). (**C**) (Simplified) gene expression network and predicted interactions. Orange interaction nodes indicate stronger activation in triple-top responders. Abbreviations: S: spike; T1: 1 month post booster; Th: T helper cells.

**Suppl. Figure S8. Support vector machine analysis to predict T6 responses based on spike (S)-specific Th-cell frequencies at T0, S-induced cytokine release at T1, and S IgG at T1.**

Abbreviations: MF: multifunctional (i.e., double/triple-positive); S: spike; spec.: specific; T0, T1, T6: 0, 1, or 6 months post booster; Th: T helper cells.

**Suppl. Figure S9. Support vector machine analysis to predict T6 responses based on spike (S)-induced cytokine release at T1.**

Abbreviations: S: spike; T1, T6: 1 or 6 months post booster; Th: T helper cells.

**Suppl. Table 1. Demographic data of study participants at each timepoint**

| **T0**  **N = 30 evaluable subjects** | |
| --- | --- |
| Median date of 1^st^ vaccination | 30.01.2021 |
| Median date of 2^nd^ vaccination | 24.02.2021 |
| Median date of 3^rd^ vaccination | 04.11.2021 |
| Median date of 1-month follow-up | 08.12.2021 |
| Median date of 3-month follow-up | 09.02.2022 |
| Median date of 6-month follow-up | 11.05.2022 |
| Median age at 3^rd^ vaccination | 51 (range 26 - 65) |
| Sex | 25 female (83.3 %), 5 male (16.7 %) |
|  |  |
| **T1**  **N = 29 evaluable subjects** | |
| Median date of 1^st^ vaccination | 27.01.2021 |
| Median date of 2^nd^ vaccination | 22.02.2021 |
| Median date of 3^rd^ vaccination | 04.11.2021 |
| Median date of 1-month follow-up | 08.12.2021 |
| Median date of 3-month follow-up | 09.02.2022 |
| Median date of 6-month follow-up | 11.05.2022 |
| Median age at 3^rd^ vaccination | 52 (range 26 - 65) |
| Sex | 24 female (82.8 %), 5 male (17.2 %) |
|  |  |
| **T3**  **N = 24 evaluable subjects** | |
| Median date of 1^st^ vaccination | 30.01.2021 |
| Median date of 2^nd^ vaccination | 24.02.2021 |
| Median date of 3^rd^ vaccination | 04.11.2021 |
| Median date of 1-month follow-up | 08.12.2021 |
| Median date of 3-month follow-up | 09.02.2022 |
| Median date of 6-month follow-up | 11.05.2022 |
| Median age at 3^rd^ vaccination | 51 (range 26 - 65) |
| Sex | 20 female (83.3 %), 4 male (16.7 %) |
|  |  |
| **T6**  **N = 16 evaluable subjects** | |
| Median date of 1^st^ vaccination | 30.01.2021 |
| Median date of 2^nd^ vaccination | 24.02.2021 |
| Median date of 3^rd^ vaccination | 04.11.2021 |
| Median date of 1-month follow-up | 08.12.2021 |
| Median date of 3-month follow-up | 09.02.2022 |
| Median date of 6-month follow-up | 11.05.2022 |
| Median age at 3^rd^ vaccination | 54 (range 35 - 63) |
| Sex | 13 female (81.3 %), 3 male (18.7 %) |

**Suppl. Table 2. Preparation of stimulation tubes.**

|  | **α-CD28** | **α-CD49d** | **SARS-CoV-2 Prot_S** | **CPI Positive Control Solution** | **CEF-MHC Class I Control Peptide Pool “Plus”** |
| --- | --- | --- | --- | --- | --- |
| **Manufacturer** | Miltenyi Biotec | Miltenyi Biotec | Miltenyi Biotec | CTL | CTL |
| **Catalog number** | 130-093-375 | 130-093-279 | 130-126-700 | CTL-CPI-001 | CTL-CEF-002 |
| **Negative control** | 10 µg/mL | 10 µg/mL | --- | --- | --- |
| **SARS-CoV-2 spike** | 10 µg/mL | 10 µg/mL | 0.6 nmol per peptide/mL | --- | --- |
| **Positive control** | 10 µg/mL | 10 µg/mL | --- | 0.3 nmol per peptide/mL | 0.3 nmol per peptide/mL |

RPMI 1640 was added to adjust reagent volume to 50 µL and 500 µL for flow cytometry and cytokine/transcriptomic tubes, respectively. A combination of CPI and CEF was used as positive control. Final concentrations provided in the table take into account the blood volume to be added. Test tubes were cryopreserved for up to 4 weeks. Manufacturers: Miltenyi Biotec, Bergisch Gladbach, Germany; Cellular Technology Limited (CTL), Shaker Heights, USA. Abbreviations: CEF: cytomegalovirus, Eppstein-Barr virus, and influenza virus peptides predominantly stimulating CD8^+^ cells; CPI: cytomegalovirus, parainfluenza virus, and influenza virus peptides predominantly stimulating CD4^+^ cells; MHC: Major histocompatibility complex; Prot_S: spike protein; RPMI 1640: Roswell Park Memorial Institute 1640 Medium.
